# Prevalence, Management, and Comorbidities of Adults With Atrial Fibrillation in the United States, 2019 to 2023

**DOI:** 10.1016/j.jacadv.2024.101330

**Published:** 2024-10-10

**Authors:** Connor G. Oltman, Taehyung P. Kim, James W.Y. Lee, John D. Lupu, Ruoqing Zhu, Issam D. Moussa

**Affiliations:** a Carle Illinois College of Medicine, University of Illinois Urbana-Champaign, Urbana, Illinois, USA; b Department of Statistics, University of Illinois Urbana-Champaign, Urbana, Illinois, USA; c Heart and Vascular Institute, Carle Health, Urbana, Illinois, USA

**Keywords:** atrial fibrillation, cardiac arrhythmia, electrophysiology, epidemiologyprevalence

## Abstract

**Background:**

Due to evolving risk factor profiles and an aging population, atrial fibrillation poses a significant public health challenge in the United States. Therefore, a contemporary and nationally representative epidemiological study is necessary to reassess atrial fibrillation’s impact on the health care system.

**Objectives:**

The purpose of the study was to provide the most current and detailed assessment of atrial fibrillation’s prevalence and management in the United States.

**Methods:**

This retrospective cohort study was performed using data from Epic’s Cosmos platform between January 1, 2019, and December 31, 2023. This is a multicenter, population-based study encompassing data from over 259 million patient records. The study cohort, 4,834,977 patients with atrial fibrillation, were identified from an initial cohort of 124,247,691 residents of the United States aged 18 years and older.

**Results:**

This study identified 4,834,977 patients with nontransient atrial fibrillation, with a mean age of 76 years; 55.43% of the patients were male, and 83.05% were non-Hispanic White. The overall prevalence of atrial fibrillation was 3.89%, where 0.26% of adults under 50 and 24.58% of those 90 and older had the condition. Geographically, rural areas reported a higher prevalence (5.29%) than urban areas (3.66%), with the Midwest, South, and Northeast experiencing higher rates compared to the Mountain and Pacific states. Roughly 30% of individuals with atrial fibrillation did not receive anticoagulant medication.

**Conclusions:**

The findings of this geographically diverse and nationally representative investigation align with recent prevalence studies, highlighting the increased burden of atrial fibrillation on the United States health care system.

Atrial fibrillation (AF) is the most common cardiac arrhythmia encountered in clinical practice. Associated with conditions such as heart failure^1^ and valvular heart disease,^2^ AF is a major cause of ischemic stroke^3^ and is strongly linked to increased mortality.^4^, ^5^, ^6^ In the United States, the prevalence of AF is projected to double between 2010 and 2030.^7^ This trend, coupled with evolving risk factor profiles^8^^,^^9^ and an aging population,^10^ positions AF as a considerable public health challenge, highlighting the need for a greater understanding of the disease's impact on the U.S. health care landscape.

Despite extensive research efforts, there remains an unmet need for a detailed and contemporary evaluation of AF's prevalence in the U.S. population. Prior investigations have primarily used administrative databases^7^^,^^11^^,^^12^ or narrowly focused but well-described cohort studies,^13^, ^14^, ^15^ with each methodology presenting its own set of unique challenges. Administrative databases might not fully capture patient comorbidities or precisely reflect clinical diagnoses, owing to variations in coding practices and billing procedures.^16^ Conversely, studies involving specific cohorts encounter challenges such as limited diversity, smaller sample sizes, and geographic constraints—factors that hinder their scalability and broader application.^17^

Electronic health records (EHR) are increasingly recognized as a foundational resource for epidemiological studies, offering a robust methodology for the investigation of medical conditions such as AF.^18^ Curated from multiple institutions, these records deliver detailed and physician-verified diagnostics to best characterize a patient's health profile. Epic Systems, a leading EHR provider in the United States,^19^ recently launched Cosmos, a clinically integrated and deidentified database designed to aggregate a wide range of clinical data in a consistent and standardized manner. This database is not only nationally representative but also clinically precise, with available data spanning patient problem lists, medications, procedures, social histories, and geographic information. Utilizing data from Cosmos spanning 2019 to 2023, this study aimed to provide the most current and detailed assessment of AF prevalence across the United States, facilitating deeper insights into AF's management and care trajectories.

## Methods

### Data source

This retrospective cohort study was performed using data from the Cosmos platform, a community collaboration of health systems representing over 259 million patient records from over 1,548 hospitals and 35,400 clinics across the United States.^20^ Cosmos contains a Health Insurance Portability and Accountability Act-defined limited dataset, and patients with records at multiple health care organizations are merged.^21^ Cosmos integrates both inpatient and outpatient charts into a single record to create a comprehensive and detailed patient health profile. The patient distribution in Cosmos closely mirrors the demographic distribution of the U.S. Census, ensuring that the findings are nationally representative and applicable across various population groups. This study was exempt from institutional review board approval and the need for informed consent in accordance with 45 CFR §46.102.

### Study population

A base patient cohort was assembled, consisting of U.S. residents aged 18 years or older who had at least 1 encounter between January 1, 2019, and December 31, 2023. Individuals were included if they appeared in Cosmos's base patient registry, a status achieved by having at least 2 face-to-face encounters within any 2-year period of their medical history. This methodology, which is standard for studies using the Cosmos database, ensured an analysis focused on longitudinal patient charts.

From the base patient cohort, individuals with AF were then identified using the International Classification of Diseases-10th Revision (ICD-10) codes described in Supplemental Table 1. In line with previous studies, patients meeting eligibility criteria for AF had ≥1 inpatient diagnosis or ≥2 outpatient diagnosis.^7^^,^^12^^,^^22^ Additionally, patients were included if they had an active diagnosis within the problem list. Diagnoses listed as resolved were included if active at any point during the specified date range, while those deleted from the problem list were excluded, as deletion signified an erroneous addition. The initial diagnosis of AF within this observation period served as the index date.

Several exclusion criteria were applied to remove cases of AF stemming from transient or reversible causes, including hyperthyroidism, binge drinking, and cardiac surgery.^23^ Patients were excluded if evidence of concomitant hyperthyroidism or alcohol/other substance abuse was noted within 12 months of the index AF diagnosis without subsequent diagnoses. Patients were also excluded if they underwent cardiac surgery (coronary artery bypass graft, pericardial surgery, structural cardiac repair surgery, and valve repair or replacement surgery) within 30 days of the index date with no evidence of later diagnoses. A detailed list of ICD-10 and Current Procedural Terminology codes used for patient exclusion can be found in Supplemental Table 2.

### Prevalence calculations

Prevalence rates were calculated as the number of AF patients divided by the total number of individuals in the base patient cohort. The overall 5-year period prevalence was computed and stratified by age, sex, race and ethnicity, U.S. Census region, rural or urban living status, socioeconomic quartile, smoking status, and family history. Patient sex was determined using legal documentation recorded in the EHR, such as a driver’s license. Race and ethnicity were identified through self-reported data in the EHR. Rural or urban living status was defined using rural-urban commuting area codes, with codes 1 to 3 classified as urban and codes 4 to 10 as rural. Using the patient’s most recent ZIP code of residence, socioeconomic quartile was derived from census tract data provided by the U.S. Centers for Disease Control and Prevention. Additionally, a heatmap was created using an online mapping tool to visualize the unadjusted prevalence of AF across U.S. counties.^24^ Counties with 10 or fewer AF patients were excluded from the analysis.

### Patient comorbidities

The base patient cohort was stratified by the presence or absence of AF and by age (working age: 18-64 years; elderly: ≥65 years). Baseline patient comorbidities included obesity (any recorded body mass index ≥30 kg/m^2^ during the observation period), hypertension, diabetes mellitus, dyslipidemia, peripheral vascular disease, chronic obstructive pulmonary disease, coronary artery disease, heart failure, chronic kidney disease, obstructive sleep apnea, prior stroke, prior myocardial infarction, valve disease, and bleeding events. Patient comorbidities were identified using ICD-10 codes, as detailed in Supplemental Table 3.

### Management strategies

This study included medications and procedures as outlined in the 2023 American College of Cardiology/American Heart Association/American College of Clinical Pharmacy/Heart Rhythm Society Guidelines for the Diagnosis and Management of Atrial Fibrillation.^23^ Medication data encompassed active prescriptions, medications documented during clinical care, and entries made by providers during medication reconciliation. The medication strategies analyzed were rate control, rhythm control, and anticoagulation. The procedures included electrical cardioversion, atrioventricular (AV) node ablation, pulmonary vein ablation, and left atrial appendage occlusion (LAAO). The ICD-10 and Current Procedural Terminology codes used to identify these procedures are detailed in Supplemental Table 4.

### Statistical analysis

Prevalence data and patient comorbidities are expressed as proportions with unadjusted ORs and 95% CIs. The chi-squared test was used to statistically compare categorical variables. Statistical significance was defined as a 2-tailed *P* value ≤0.05. All statistical analyses were performed using Microsoft Excel.^25^

## Results

### Prevalence of atrial fibrillation

**Figure 1:**
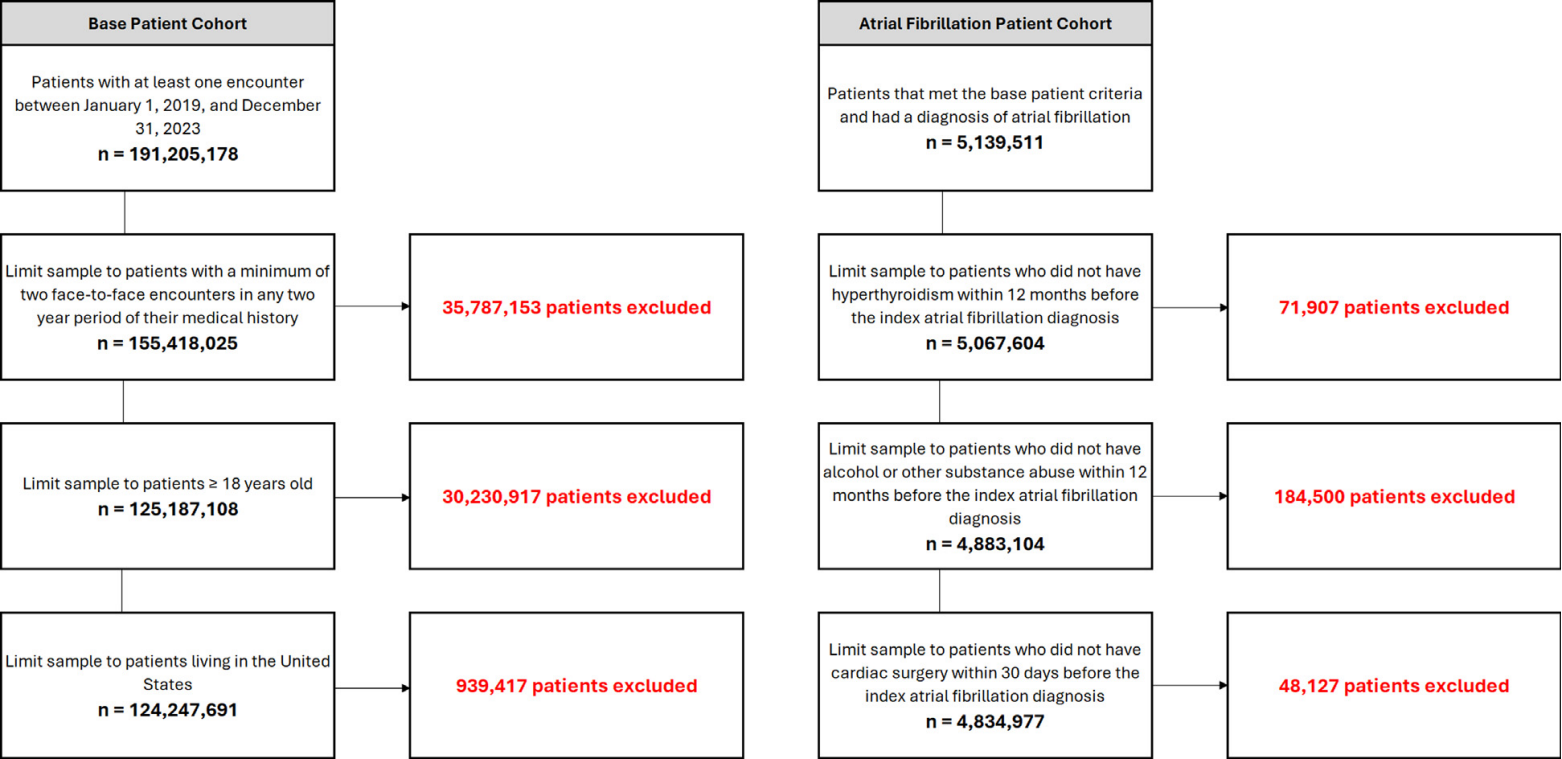
**Design of the Base Patient and Atrial Fibrillation Cohorts** From the base patient cohort, individuals with nontransient atrial fibrillation were identified by at least 1 inpatient diagnosis or 2 outpatient diagnoses during the observation period, or a diagnosis on their problem list. Patients with a solitary atrial fibrillation diagnosis due to a transient cause, such as hyperthyroidism, binge drinking, or recent cardiac surgery, were excluded from the analysis.

This study identified 4,834,977 patients with nontransient AF, with an overall mean age of 76 ± 12 years; 55.43% of the patients were male, and 83.05% were non-Hispanic (NH) White. This cohort was derived from a broader group of 124,247,691 patients who met the initial screening criteria (Figure 1). Consequently, the overall 5-year prevalence of AF from 2019 to 2023 was calculated to be 3.89%.

The prevalence of AF exhibited substantial demographic variability, with males demonstrating a higher overall prevalence at 4.87% compared to females at 3.11%. Stratifying by race and ethnicity revealed that NH White individuals had the highest AF prevalence at 5.30%, followed by NH American Indian and Alaskan Native individuals at 3.17%, NH Native Hawaiian and Other Pacific Islanders at 2.46%, NH Black or African American patients at 2.17%, NH Asian patients at 1.67%, and Hispanic patients at 1.18%. Geographically, rural areas reported a higher AF prevalence at 5.29% than urban locales at 3.66%. Detailed prevalence data are presented in Table 1.

**Table 1:** Prevalence of Atrial Fibrillation by Age, Sex, Racial and Ethnic Group, Geographic Region, Socioeconomic Quartile, Smoking Status, and Family History

	All Patients	AF Patients	Prevalence of AF (%)	uOR (95% CI)
Age range, y				
<50	58,672,830	149,788	0.26	1.00 (Ref)
50-59	18,944,409	289,319	1.53	9.90 (9.86-9.94)
60-69	20,573,254	838,611	4.08	29.59 (29.52-29.66)
70-79	15,671,591	1,482,928	9.46	85.78 (85.61-85.94)
80-89	7,778,166	1,422,447	18.29	174.68 (174.33-175.03)
≥90	2,607,441	640,884	24.58	199.51 (198.93-200.08)
Sex				
Female	69,191,753	2,154,341	3.11	1.00 (Ref)
Male	54,983,864	2,680,210	4.87	2.45 (2.45-2.45)
Race and ethnicity				
NH White	75,810,070	4,015,603	5.30	1.00 (Ref)
NH Black or African American	16,863,332	365,987	2.17	0.17 (0.17-0.17)
Hispanic	13,525,159	160,244	1.18	0.09 (0.09-0.09)
NH Asian	4,767,121	79,433	1.67	0.13 (0.13-0.13)
NH American Indian or Alaska Native	744,344	23,621	3.17	0.26 (0.26-0.26)
NH Native Hawaiian and Other Pacific Islander	464,836	11,419	2.46	0.20 (0.20-0.21)
United States census region				
West	19,103,726	525,920	2.75	1.00 (Ref)
Midwest	31,542,555	1,384,607	4.39	1.76 (1.75-1.76)
Northeast	25,432,548	1,081,245	4.25	1.67 (1.66-1.67)
South	47,674,820	1,838,997	3.86	1.47 (1.47-1.47)
Rural or urban living status				
Urban	105,295,219	3,851,821	3.66	1.00 (Ref)
Rural	17,804,553	941,103	5.29	2.05 (2.04-2.05)
Socioeconomic quartile				
1st	31,839,291	1,337,985	4.20	1.00 (Ref)
2nd	27,332,677	1,166,593	4.27	1.02 (1.01-1.02)
3rd	28,067,705	1,158,280	4.13	0.97 (0.97-0.97)
4th	35,539,550	1,116,441	3.14	0.67 (0.67-0.68)
Smoking status				
Never	69,224,359	2,355,946	3.40	1.00 (Ref)
Current or former	38,499,127	2,284,210	5.93	2.64 (2.64-2.64)
Family history of AF				
No	123,449,683	4,715,435	3.82	1.00 (Ref)
Yes	798,008	119,542	14.98	15.38 (15.29-15.48)

AF = atrial fibrillation; NH = non-Hispanic; uOR = unadjusted OR.

The heatmap identified Nebraska as the state with the highest prevalence, at 6.94%, with Maine and Rhode Island following at 5.50% and 4.99%, respectively. At the opposite end of the spectrum, Utah, Colorado, and Nevada presented the lowest state prevalence rates, at 1.60%, 1.83%, and 1.91%, respectively. The data delineate clear regional patterns of AF prevalence, with the Midwest, Southern, and Northeastern states showing a higher prevalence compared to the Mountain and Pacific states, as depicted in the Central Illustration.

### Patient comorbidities

Among AF patients, notable demographic findings include an elevated burden of baseline comorbidities, including hypertension, diabetes, dyslipidemia, chronic kidney disease, chronic obstructive lung disease, peripheral vascular disease, and obstructive sleep apnea, in the AF cohort compared to those without AF (Table 2). Additionally, comorbid cardiac conditions such as heart failure, coronary artery disease, myocardial infarction, and valve disease were more prevalent among individuals with AF. Bleeding events and ischemic stroke were also seen more frequently in AF patients. Comorbidity OR were higher in the 18- to 64-year-old group than those observed for the ≥65-year-old group. Heart failure emerged as the comorbidity with the highest OR in both age groups.

**Table 2:** Patient Comorbidities Stratified by Age Group and Presence or Absence of Atrial Fibrillation^a^

	Working Age (18-64 Years of Age)			Elderly (≥65 Years of Age)		
	AF Not Observed (n = 91,547,061)	AF Observed (n = 857,423)	uOR (95% CI)	AF Not Observed (n = 27,865,653)	AF Observed (n = 3,977,554)	uOR (95% CI)
Obesity^b^	36,319,231 (39.67)	572,061 (66.72)	3.05 (3.04-3.06)	9,796,028 (35.15)	1,914,691 (48.14)	1.71 (1.71-1.72)
Hypertension	21,349,611 (23.32)	670,300 (78.18)	11.78 (11.74-11.82)	16,555,384 (59.41)	3,552,967 (89.33)	5.72 (5.71-5.73)
Diabetes	8,836,272 (9.65)	299,801 (34.97)	5.03 (5.01-5.05)	6,663,006 (23.91)	1,461,294 (36.74)	1.85 (1.84-1.85)
Dyslipidemia	21,089,229 (23.04)	594,528 (69.34)	7.56 (7.53-7.58)	15,676,505 (56.26)	3,169,062 (79.67)	3.05 (3.04-3.05)
Chronic kidney disease	2,569,607 (2.81)	182,080 (21.24)	9.34 (9.29-9.38)	4,289,457 (15.39)	1,496,437 (37.62)	3.31 (3.31-3.32)
Chronic obstructive lung disease	2,705,271 (2.96)	154,419 (18.01)	7.21 (7.17-7.25)	3,174,851 (11.39)	975,742 (24.53)	2.53 (2.52-2.53)
Peripheral vascular disease	1,408,574 (1.54)	83,445 (9.73)	6.90 (6.85-6.95)	1,830,553 (6.57)	628,387 (15.80)	2.67 (2.66-2.68)
Coronary artery disease	4,050,918 (4.42)	327,556 (38.20)	13.35 (13.30-13.41)	5,635,529 (20.22)	2,041,058 (51.31)	4.16 (4.15-4.17)
Heart failure	1,696,753 (1.85)	284,724 (33.21)	26.33 (26.21-26.45)	2,485,467 (8.92)	1,844,196 (46.37)	8.83 (8.81-8.85)
Obstructive sleep apnea	5,498,430 (6.01)	305,130 (35.59)	8.65 (8.61-8.68)	2,694,787 (9.67)	939,494 (23.62)	2.89 (2.88-2.90)
Ischemic Stroke	1,027,563 (1.12)	81,950 (9.56)	9.31 (9.24-9.38)	1,302,208 (4.67)	545,608 (13.72)	3.24 (3.23-3.25)
Myocardial infarction	1,063,393 (1.16)	96,585 (11.26)	10.80 (10.73-10.88)	1,191,157 (4.27)	532,886 (13.40)	3.46 (3.45-3.48)
Valve disease						
Aortic stenosis	236,263 (0.26)	34,932 (4.07)	16.41 (16.23-16.60)	767,488 (2.75)	437,475 (11.00)	4.36 (4.35-4.38)
Aortic regurgitation	320,521 (0.35)	38,415 (4.48)	13.35 (13.21-13.49)	551,450 (1.98)	287,138 (7.22)	3.85 (3.84-3.87)
Mitral regurgitation	861,648 (0.94)	126,818 (14.79)	18.27 (18.15-18.38)	1,088,222 (3.91)	777,629 (19.55)	5.98 (5.96-6.00)
Tricuspid regurgitation	413,781 (0.45)	50,412 (5.88)	13.76 (13.63-13.89)	442,591 (1.59)	320,470 (8.06)	5.43 (5.40-5.45)
Bleeds						
Intracranial hemorrhage	372,251 (0.41)	17,392 (2.03)	5.07 (4.99-5.15)	365,687 (1.31)	135,647 (3.41)	2.66 (2.64-2.67)
Gastrointestinal hemorrhage	3,177,713 (3.47)	81,109 (9.46)	2.91 (2.88-2.93)	1,593,387 (5.72)	480,988 (12.09)	2.27 (2.26-2.28)

Values are n (%) unless otherwise indicated.

AF = atrial fibrillation; uOR = unadjusted OR.

a All *P* < 0.001.

b Includes patients with a body mass index of 30 kg/m^2^ or higher at any point during the study period.

### Management strategies

**Figure 2:**
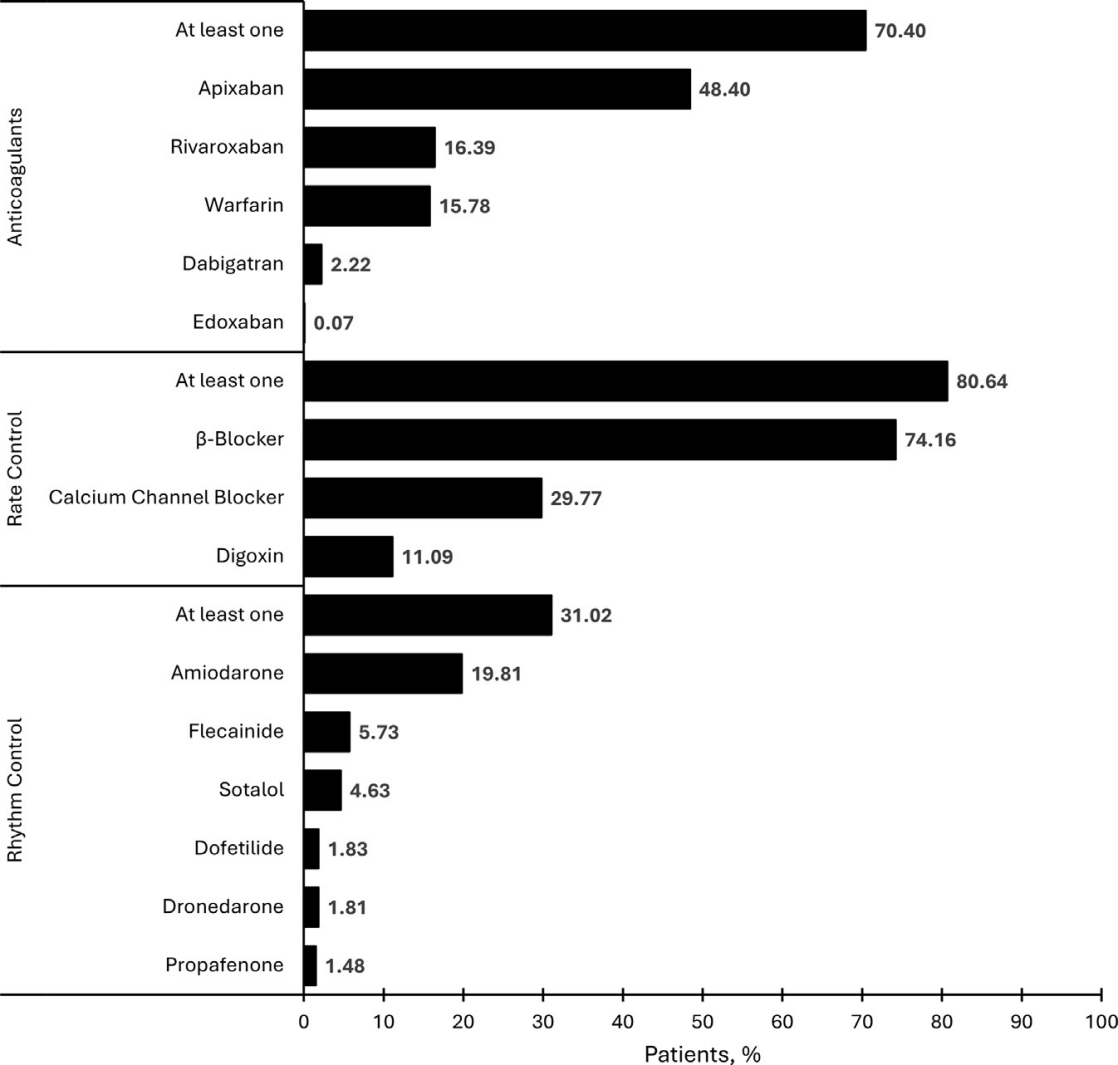
**Rate Control, Rhythm Control, and Anticoagulant Medication Use Among Patients With Atrial Fibrillation** Medication data encompassed active prescriptions, medications documented during clinical care, and entries made by providers during medication reconciliation. All medications listed are outlined in the 2023 American College of Cardiology/American Heart Association/American College of Clinical Pharmacy/Heart Rhythm Society guidelines for the diagnosis and management of atrial fibrillation.^23^ Calcium-channel blockers include verapamil and diltiazem, and β-blockers include metoprolol, atenolol, bisoprolol, carvedilol, esmolol, nadolol, and propranolol.

**Figure 3:**
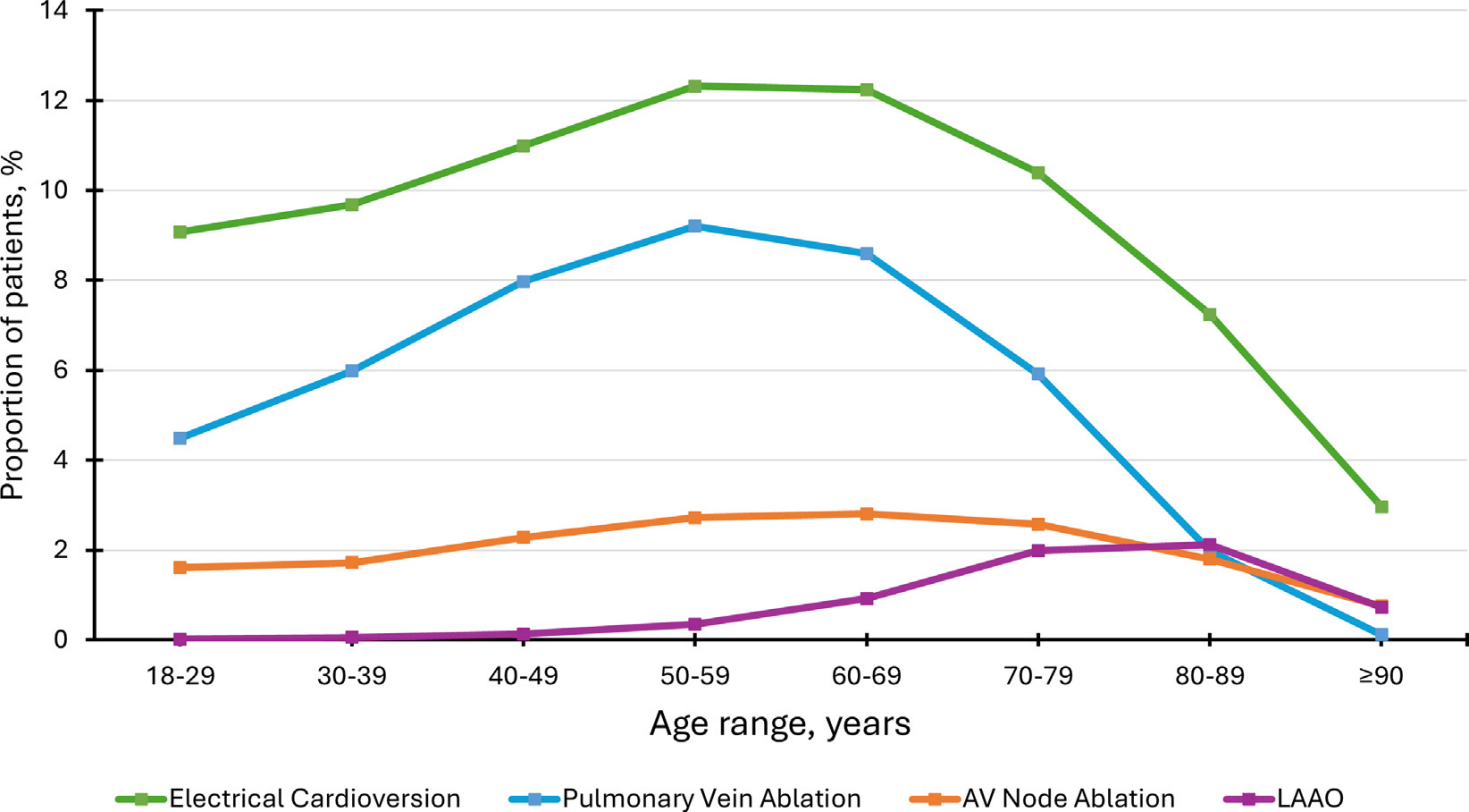
**Elective Procedure Use Among Patients With Atrial Fibrillation, Stratified by Age** The graph illustrates the utilization of electrical cardioversion, pulmonary vein ablation, AV node ablation, and LAAO across different age groups, highlighting treatment trends in the management of atrial fibrillation. Cardioversion emerged as the preferred approach for acute rhythm control, likely due to its prompt effectiveness. Pulmonary vein ablation was performed at a frequency more than twice that of AV node ablation. Older adults received LAAO more frequently than younger patients. AV = atrioventricular; LAAO = left atrial appendage occlusion.

In the AF cohort, 70.40% of patients received at least 1 anticoagulant prescription, predominantly Apixaban (Figure 2). Rate control medication was prescribed to 80.64% of the cohort, with beta-blockers being the most frequently selected option. Amiodarone was the leading choice for rhythm control, where 31.02% of patients received at least 1 medication. In terms of elective procedures, 8.91% of AF patients received electrical cardioversion, 4.68% received pulmonary vein ablation, 2.14% underwent AV node ablation, and 1.52% underwent LAAO. Elective procedures used in the AF cohort were also segmented by age (Figure 3).

**Central Illustration:**
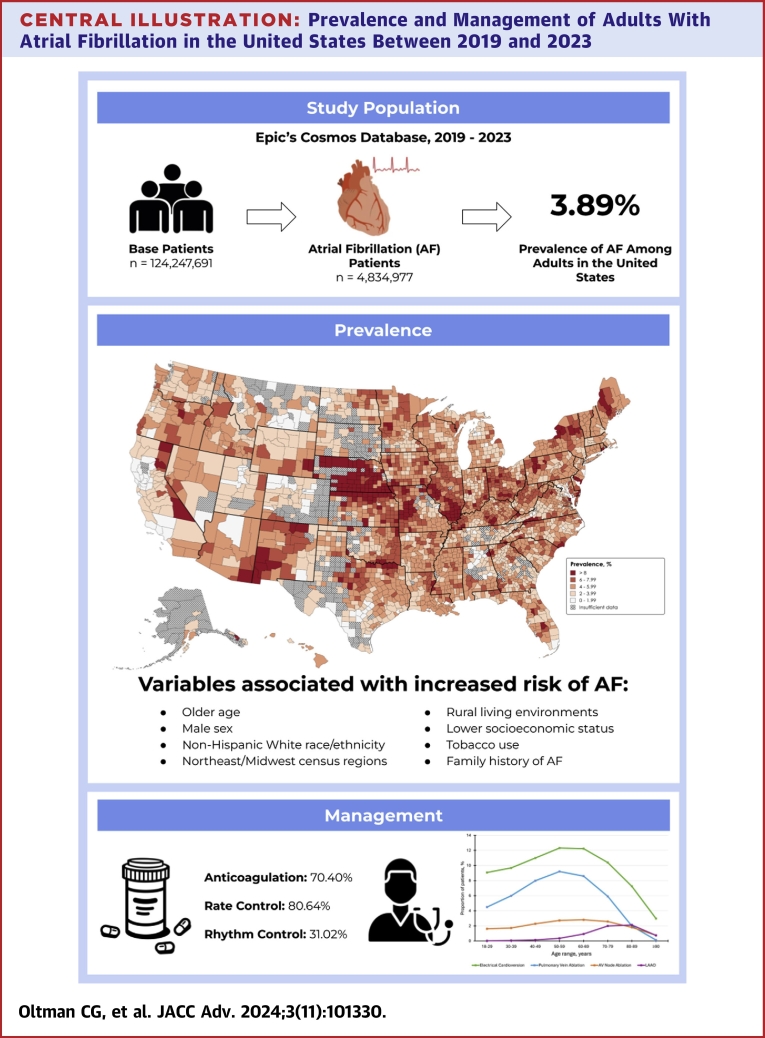
**Prevalence and Management of Adults With Atrial Fibrillation in the United States Between 2019 and 2023** Data from Epic’s Cosmos platform reveal an increased prevalence of AF in the United States, particularly among older adults, males, non-Hispanic White patients, individuals living in rural areas, those with low socioeconomic status, tobacco smokers, and those with a family history of atrial fibrillation. Rate control remains the dominant strategy for managing AF, despite the recent increase in the use of rhythm control strategies. Additionally, a significant proportion of patients with AF are not receiving anticoagulation medications, highlighting a critical gap in clinical practice. AF = atrial fibrillation; AV = atrioventricular.

## Discussion

This study identified a heightened prevalence of AF, consistent with recent observations, while providing a more comprehensive evaluation of its epidemiology across the United States. The analysis revealed substantial regional and demographic disparities in AF prevalence, with specific populations facing a higher burden of disease. Additionally, this study revealed a persistent gap in care, particularly the underutilization of anticoagulation therapy, highlighting a critical area for clinical improvement. These findings are crucial for informing clinical practice by identifying high-risk populations and guiding interventions aimed at reducing stroke risk and improving AF management across diverse patient groups.

Historical estimates of AF prevalence have varied significantly, likely influenced by the demographic characteristics of the study populations and the methods used for detection. However, most estimates fall between 1% and 2.5%.^26^, ^27^, ^28^ Against this backdrop, the overall prevalence of 3.89% found in this study represents a notable deviation from the conventional range. Recent research supports this result; a 2022 analysis from the All of Us Research Program reported a 4.2% prevalence among U.S. adults with accessible EHR data.^29^ Additionally, a 2023 prospective study conducted in Massachusetts revealed an AF prevalence of 13.2% in adults aged 65 years and over, a finding comparable to the 12.49% prevalence observed in this study for the same age group.^30^

This study found that the prevalence of AF increased with age, male sex, and NH White race/ethnicity, aligning with prior publications.^29^^,^^30^ Additionally, AF was more prevalent among patients with low socioeconomic status, a family history of AF, and tobacco use—risk factors extensively documented in previous studies.^10^

Despite the documented higher prevalence of AF among NH White patients, the underlying reasons for this disparity warrant thorough consideration. Factors such as ascertainment bias and access to health care play crucial roles. Non-White groups often face significant barriers to regular health care access, leading to underdetection and underdiagnosis of AF. Additionally, survival bias may contribute to these findings, as the longer life expectancy among NH White populations correlates with an increased likelihood of AF detection over time.^31^ Furthermore, the asymptomatic presentation of AF can obscure its prevalence among individuals who do not regularly utilize health care services, resulting in a lower reported prevalence in these groups despite potentially comparable actual rates. These considerations highlight the importance of equitable health care access to accurately identify and manage AF across diverse populations.

The geographical distribution of AF prevalence across U.S. counties, as highlighted in the map, offered intriguing insights. While the Southern states have reported higher incidences of risk factors such as hypertension, obesity, and diabetes,^32^ the Midwest and Northeast demonstrate elevated AF prevalence—a phenomenon that may be influenced by racial demographics and access to health care in these regions. Predominantly NH White populations^33^ and enhanced health care utilization in the Midwest and Northeast^34^ may contribute to more frequent AF detection. Meanwhile, the Western states present a reduced disease burden, aligning with broader health trends that could be ascribed to healthier lifestyle choices, higher average income, and a strong emphasis on proactive public health measures. Additionally, the observed variation in AF prevalence between urban and rural locales may reflect the older demographic prevalent in rural areas.^35^ To better understand these disparities, future research should analyze treatment and management variations among AF patients across different regions of the United States.

### Patient comorbidities

In this study, baseline comorbidities such as hypertension, diabetes, and dyslipidemia were more common among AF patients. Conditions including heart failure, coronary artery disease, valvular heart disease, and myocardial infarction exhibited high OR in both younger and older populations, underlining the significant impact of AF on overall cardiac health. The link between AF and bleeding events may be due to the adverse effects associated with anticoagulant medication use.

Comorbidity OR were greater among AF patients aged 18 to 64 years compared to those aged 65 years and older, underscoring the significance of AF as a health marker in younger patients. While elderly individuals with AF may have health outcomes comparable to their peers without AF, the presence of AF in patients under 65 years typically indicates substantial underlying comorbidities. Therefore, managing AF in this younger demographic necessitates more vigilant monitoring and intervention.

### Medications and procedures

Utilization of anticoagulants in this study aligns with recent findings. In 2020, anticoagulant use among AF patients was reported at 64.7%, compared to the 70.4% of patients who received these medications in this study.^36^ Additionally, warfarin use was reported at 17.7%, which decreased to 15.78% in this study.^36^ This shift highlights a growing preference for newer anticoagulants over warfarin, likely due to their more favorable side effect profiles.^37^ This study also highlighted a significant public health concern: a high rate (29.60%) of AF patients under active medical care do not receive any anticoagulant, thereby increasing their risk of ischemic stroke and its known sequalae of disability and mortality. It is unlikely that 1 out of every 3 AF patients has a contraindication to anticoagulants. This care gap necessitates national efforts to establish benchmarks for anticoagulant use in AF patients, akin to the successful campaigns that reduced door-to-balloon times for patients with ST elevation myocardial infarction.^38^

Historically, rate control has been a dominant strategy in managing AF, with rhythm control reserved for cases where symptoms persist despite effective rate management.^23^ This study reveals a marked increase in the adoption of rhythm control strategies compared to earlier studies on antiarrhythmic medication use.^39^ In 2000, 56.2% of AF patients were prescribed rate-control medications; in contrast, 80.64% of patients in this study received such treatments, indicating a growth of 43%. Conversely, the use of rhythm control strategies rose from 12.2% to 31.02%, an increase of 154%. This trend reflects the current shift in the treatment paradigm for AF, favoring the early initiation of rhythm management strategies.^40^

Regarding elective procedures, cardioversion emerged as the preferred approach for acute rhythm control in AF, likely due to its prompt effectiveness. Pulmonary vein ablation, favored for its higher success rate,^41^ was performed at a frequency more than twice that of AV node ablation. The use of LAAO in older adults may be attributed to concerns about the bleeding risks associated with oral anticoagulant therapy, given that the incidence of major bleeding events tends to increase with age.^42^

### Strengths and limitations

This study leverages the strengths inherent to the Cosmos database. This vast and diverse dataset not only ensures a large sample size but also guarantees the heterogeneity necessary for a representation reflective of the U.S. population. This breadth permits a nuanced analysis that is both broad in scope and rich in detail, allowing for the examination of AF management across varied demographic, geographic, and clinical contexts. The reliance on physician-entered diagnoses and treatment decisions within this integrated database further enhances the study's accuracy and relevance, enabling access to comprehensive details on patient medications, procedures, social histories, and geographic locations from a singular, unified source.

However, the Cosmos database also has limitations, including the potential for data entry errors and inconsistencies in the use of problem lists. Even with stringent quality control measures in place, including compliance standards set by The Joint Commission and internal quality improvement audits, the elimination of data entry inaccuracies by providers is not feasible.^43^ These inaccuracies could impact the completeness and fidelity of EHR data. Additionally, the inconsistent use of problem lists across different health care institutions introduces further variability, potentially affecting the uniformity of data across various settings.^44^

The design of the Cosmos platform, which excludes queries with 10 or fewer patients, restricts this study's ability to evaluate AF prevalence in areas with minimal documentation. This limitation, in conjunction with the predominance of Epic's EHR system in medium- to large-sized hospitals (due to costs associated with implementation), likely introduces a bias toward data from urban or suburban health facilities.^19^ Consequently, data from smaller health care units or primary care physicians may be underrepresented. Additionally, patients with asymptomatic AF who do not regularly interact with the health care system may be overlooked. As a result, this study may inadequately reflect AF prevalence in rural or sparsely populated areas, thus limiting the comprehensiveness of the geographic analysis.

Finally, due to the lack of access to line-level patient data, the heatmap of AF prevalence by U.S. county could not be adjusted for age, sex, or race/ethnicity. This limitation extends to the OR associated with the comorbidity and prevalence data. Consequently, the inability to control for potential confounders may result in inflated county-level prevalence rates and OR. Despite this limitation, the overall size of the database provides a comprehensive look at the prevalence landscape of AF in the United States, offering valuable insights into its epidemiology and management across diverse populations.

## Conclusions

Leveraging the novel research capabilities of the Cosmos database, this study has unveiled epidemiological patterns of AF that resonate with current findings in the field. With its unprecedented size and representativeness, the Cosmos database offers the most current insights into AF prevalence within the U.S. adult population. This investigation not only establishes a new benchmark for AF prevalence studies but also highlights the critical role of large-scale, comprehensive data sources in advancing cardiovascular health research.

Perspectives **COMPETENCY IN MEDICAL KNOWLEDGE:** By providing detailed and contemporary prevalence data, this study equips clinicians with a comprehensive understanding of the epidemiological landscape of AF. Additionally, this investigation elucidates national-level AF management strategies, enabling clinicians to evaluate and align their practices with established standards across the country.

**TRANSLATIONAL OUTLOOK:** This study confirms that the prevalence of AF is rising in the U.S., particularly among older adults. As risk factors become more prevalent and the population continues to age, proactive efforts are required to minimize the burden of AF on the healthcare system. With nearly 30% of AF patients not receiving anticoagulation medication, there exists a critical gap in clinical practice; systematic efforts are required to reduce stroke risk via appropriate anticoagulant use. Additionally, further research is needed to understand why some patients with AF do not receive anticoagulation medication.

## Funding support and author disclosures

The authors have reported that they have no relationships relevant to the contents of this paper to disclose.

## References

[1] Anter E., Jessup M., Callans D.J. (2009). Atrial fibrillation and heart failure: treatment considerations for a dual epidemic. Circulation.

[2] Darby A.E., Dimarco J.P. (2012). Management of atrial fibrillation in patients with structural heart disease. Circulation.

[3] Wolf P.A., Abbott R.D., Kannel W.B. (1991). Atrial fibrillation as an independent risk factor for stroke: the Framingham Study. Stroke.

[4] Sankaranarayanan R., Kirkwood G., Visweswariah R., Fox D.J. (2015). How does chronic atrial fibrillation influence mortality in the modern treatment era?. Curr Cardiol Rev.

[5] John R.M., Michaud G.F., Stevenson W.G. (2018). Atrial fibrillation hospitalization, mortality, and therapy. Eur Heart J.

[6] Garside T., Bedford J.P., Vollam S., Gerry S., Rajappan K., Watkinson P.J. (2022). Increased long-term mortality following new-onset atrial fibrillation in the intensive care unit: a systematic review and meta-analysis. J Crit Care.

[7] Colilla S., Crow A., Petkun W., Singer D.E., Simon T., Liu X. (2013). Estimates of current and future incidence and prevalence of atrial fibrillation in the U.S. adult population. Am J Cardiol.

[8] Schnabel R.B., Yin X., Gona P. (2015). 50 year trends in atrial fibrillation prevalence, incidence, risk factors, and mortality in the Framingham Heart Study: a cohort study. Lancet.

[9] Lakshminarayan K., Anderson D.C., Herzog C.A., Qureshi A.I. (2008). Clinical epidemiology of atrial fibrillation and related cerebrovascular events in the United States. Neurol.

[10] Kornej J., Börschel C.S., Benjamin E.J., Schnabel R.B. (2020). Epidemiology of atrial fibrillation in the 21st century: novel methods and new insights. Circ Res.

[11] Piccini J.P., Hammill B.G., Sinner M.F. (2012). Incidence and prevalence of atrial fibrillation and associated mortality among Medicare beneficiaries, 1993-2007. Circ Cardiovasc Qual Outcomes.

[12] Turakhia M.P., Shafrin J., Bognar K. (2018). Estimated prevalence of undiagnosed atrial fibrillation in the United States. PLoS One.

[13] Wolf P.A., Benjamin E.J., Belanger A.J., Kannel W.B., Levy D., D'Agostino R.B. (1996). Secular trends in the prevalence of atrial fibrillation: the Framingham Study. Am Heart J.

[14] Psaty B.M., Manolio T.A., Kuller L.H. (1997). Incidence of and risk factors for atrial fibrillation in older adults. Circulation.

[15] Alonso A., Agarwal S.K., Soliman E.Z. (2009). Incidence of atrial fibrillation in whites and african-Americans: the atherosclerosis risk in communities (ARIC) study. Am Heart J.

[16] Tyree P.T., Lind B.K., Lafferty W.E. (2006). Challenges of using medical insurance claims data for utilization analysis. Am J Med Qual.

[17] St Sauver J.L., Grossardt B.R., Leibson C.L., Yawn B.P., Melton LJ 3rd, Rocca W.A. (2012). Generalizability of epidemiological findings and public health decisions: an illustration from the Rochester Epidemiology Project. Mayo Clin Proc.

[18] Casey J.A., Schwartz B.S., Stewart W.F., Adler N.E. (2016). Using electronic health records for population health research: a review of methods and applications. Annu Rev Publ Health.

[19] Koppel R., Lehmann C.U. (2015). Implications of an emerging EHR monoculture for hospitals and healthcare systems. J Am Med Inf Assoc.

[20] Tarabichi Y., Frees A., Honeywell S. (2021). The Cosmos collaborative: a vendor-facilitated electronic health record data aggregation platform. ACI open.

[21] Son M., Gallagher K., Lo J.Y. (2021). Coronavirus disease 2019 (COVID-19) pandemic and pregnancy outcomes in a U.S. Population. Obstet Gynecol.

[22] Turakhia M.P., Guo J.D., Keshishian A. (2023). Contemporary prevalence estimates of undiagnosed and diagnosed atrial fibrillation in the United States. Clin Cardiol.

[23] Joglar J.A., Chung M.K., Armbruster A.L. (2024). 2023 ACC/AHA/ACCP/HRS guideline for the diagnosis and management of atrial fibrillation: a report of the American college of cardiology/American heart association Joint committee on clinical practice Guidelines. Circulation.

[24] Create your own custom map. MapChart. https://www.mapchart.net/.

[25] (2024). Microsoft Excel for Microsoft 365 MSO. Version 2408, Build 16.0.17928.20114.

[26] Go A.S., Hylek E.M., Phillips K.A. (2001). Prevalence of diagnosed atrial fibrillation in adults: national implications for rhythm management and stroke prevention: the AnTicoagulation and Risk Factors in Atrial Fibrillation (ATRIA) Study. JAMA.

[27] Naccarelli G.V., Varker H., Lin J., Schulman K.L. (2009). Increasing prevalence of atrial fibrillation and flutter in the United States. Am J Cardiol.

[28] Miyasaka Y., Barnes M.E., Gersh B.J. (2006). Secular trends in incidence of atrial fibrillation in Olmsted County, Minnesota, 1980 to 2000, and implications on the projections for future prevalence. Circulation.

[29] Alonso A., Alam A.B., Kamel H. (2022). Epidemiology of atrial fibrillation in the All of us research Program. PLoS One.

[30] Khurshid S., Ashburner J.M., Ellinor P.T. (2023). Prevalence and incidence of atrial fibrillation among older primary care patients. JAMA Netw Open.

[31] Thomas K.L., Garg J., Velagapudi P. (2022). Racial and ethnic disparities in arrhythmia care: a call for action. Heart Rhythm.

[32] Akinyemiju T., Jha M., Moore J.X., Pisu M. (2016). Disparities in the prevalence of comorbidities among US adults by state Medicaid expansion status. Prev Med.

[33] (2021). Racial and Ethnic Diversity in the United States: 2010 Census and 2020 Census.

[34] Black L.I., Schiller J.S. (2016). State variation in health care service utilization. United States, 2014. NCHS Data Brief.

[35] Cohen S.A., Greaney M.L. (2023). Aging in rural communities. Curr Epidemiol Rep.

[36] Navar A.M., Kolkailah A.A., Overton R. (2022). Trends in oral anticoagulant use among 436 864 patients with atrial fibrillation in community practice, 2011 to 2020. J Am Heart Assoc.

[37] Vinogradova Y., Coupland C., Hill T., Hippisley-Cox J. (2018). Risks and benefits of direct oral anticoagulants versus warfarin in a real world setting: cohort study in primary care. BMJ.

[38] Bradley E.H., Nallamothu B.K., Herrin J. (2009). National efforts to improve door-to-balloon time results from the Door-to-Balloon Alliance. J Am Coll Cardiol.

[39] Fang M.C., Stafford R.S., Ruskin J.N., Singer D.E. (2004). National trends in antiarrhythmic and antithrombotic medication use in atrial fibrillation. Arch Intern Med.

[40] Camm A.J., Naccarelli G.V., Mittal S. (2022). The increasing role of rhythm control in patients with atrial fibrillation: JACC state-of-the-art review. J Am Coll Cardiol.

[41] Khan M.N., Jaïs P., Cummings J. (2008). Pulmonary-vein isolation for atrial fibrillation in patients with heart failure. N Engl J Med.

[42] Torn M., Bollen W.L., van der Meer F.J., van der Wall E.E., Rosendaal F.R. (2005). Risks of oral anticoagulant therapy with increasing age. Arch Intern Med.

[43] Hong C.J., Kaur M.N., Farrokhyar F., Thoma A. (2015). Accuracy and completeness of electronic medical records obtained from referring physicians in a Hamilton, Ontario, plastic surgery practice: a prospective feasibility study. Plast Surg (Oakv).

[44] Wang E.C., Wright A. (2020). Characterizing outpatient problem list completeness and duplications in the electronic health record. J Am Med Inf Assoc.

