# Supplementary material for: Prevalence, Management, and Comorbidities of Adults With Atrial Fibrillation in the United States, 2019 to 2023
Source: JACC Adv. 2024 Oct 10;3(11):101330. doi: 10.1016/j.jacadv.2024.101330 (PMC11686049; doi:10.1016/j.jacadv.2024.101330)
Supplement: Supplementary material [file mmc1.docx]

**Supplemental Table 1:** ICD-10-CM codes used to define a diagnosis of AF

| **ICD-10-CM Code** | **Diagnosis** |
| --- | --- |
| I48.91 | Unspecified atrial fibrillation |
| I48.20 | Chronic atrial fibrillation |
| I48.19 | Persistent atrial fibrillation |
| I48.0 | Paroxysmal atrial fibrillation |
| I48.21 | Permanent atrial fibrillation |
| I48.11 | Longstanding atrial fibrillation |

**Supplemental Table 2:** Medical claims codes used to define exclusionary cases of AF

| **Condition/Procedure** | **Codes** | **Duration** |
| --- | --- | --- |
| Alcohol or other substance abuse | **ICD-10-CM:** F10.1xx, except F10.11  F10.2xx, except F10.21  F10.9xx, except F10.91  F11.2xx, except F11.21  F15.2xx, except F15.21  O99.31x | Within 12 months before index AF diagnosis |
| Hyperthyroidism | **ICD-10-CM:** E05.xx  **Other**: Prescription of propylthiouracil or methimazole | Within 12 months before index AF diagnosis |
| **Cardiac surgery:** coronary artery bypass graft | **ICD-10-PCS:** 0210083, 0210093, 02100A3, 02100J3, 02100K3, 02100Z3, 0210483, 0210493, 02104A3, 02104J3, 02104K3, 02104Z3  **CPT:** 33509-33514, 33516, 35600, 33533-33536 | Within 30 days before index AF diagnosis |
| **Cardiac surgery:** pericardial surgery | **ICD-10-PCS:**  02N60ZZ, 02N63ZZ, 02N64ZZ, 02N70ZZ, 02N73ZZ, 02N74ZZ, 02NK0ZZ, 02NK3ZZ, 02NK4ZZ, 02NL0ZZ, 02NL3ZZ, 02NL4ZZ, 02C60ZZ, 02C63ZZ, 02C64ZZ, 02C70ZZ, 02C73ZZ, 02C74ZZ, 02C80ZZ, 02C83ZZ, 02C84ZZ, 02C90ZZ, 02C93ZZ, 02C94ZZ, 02CK0ZZ, 02CK3ZZ, 02CK4ZZ, 02CL0ZZ, 02CL3ZZ, 02CL4ZZ, 02PA0YZ, 02PA4YZ, 02WA0YZ, 02WA3YZ, 02WA4YZ, 02CN0ZZ, 02CN3ZZ, 02CN4ZZ, 02NN0ZZ, 02NN3ZZ, 02NN4ZZ, 0W9D00Z, 0W9D0ZX, 0W9D0ZZ, 0WCD0ZZ, 0WCD3ZZ, 0WCD4ZZ, 02BN0ZX, 02BN3ZX, 02BN4ZX, 02BN0ZZ, 02BN3ZZ, 02BN4ZZ, 02TN0ZZ, 02TN3ZZ, 02TN4ZZ, 02B60ZZ, 02B63ZZ, 02B64ZZ, 02B70ZZ, 02B73ZZ, 02B74ZZ, 02BK0ZZ, 02BK3ZZ, 02BK4ZZ, 02BL0ZZ, 02BL3ZZ, 02BL4ZZ, 02560ZZ, 02570ZZ, 025K0ZZ, 025L0ZZ, 02B60ZZ, 02B70ZZ, 02BK0ZZ, 02BL0ZZ, 02T80ZZ, 028D0ZZ, 028D3ZZ, 028D4ZZ, 02QD0ZZ, 02QD3ZZ, 02QD4ZZ, 02890ZZ, 02893ZZ, 02894ZZ, 02Q90ZZ, 02Q93ZZ, 02Q94ZZ, 02QF0ZZ, 02QF3ZZ, 02QF4ZZ, 02QG0ZZ, 02QG3ZZ, 02QG4ZZ, 02QH0ZZ, 02QH3ZZ, 02QH4ZZ, 02QJ0ZZ, 02QJ3ZZ, 02QJ4ZZ, 02QA0ZZ, 02QA3ZZ, 02QA4ZZ, 02B50ZZ, 02B53ZZ, 02B54ZZ  **CPT:** 33020, 33025, 33030, 33031, 33050 | Within 30 days before index AF diagnosis |
| **Cardiac surgery:** structural cardiac repair surgery | **ICD-10-PCS:**  02RM0JZ, 02RM4JZ, 02U50JZ, 02U53JZ, 02U54JZ, 02UM0JZ, 02UM3JZ, 02UM4JZ, 024G0J2, 024J0J2, 02RM07Z, 02RM0KZ, 02RM47Z, 02RM4KZ, 02U507Z, 02U508Z, 02U50KZ, 02U537Z, 02U538Z, 02U53KZ, 02U547Z, 02U548Z, 02U54KZ, 02UM07Z, 02UM0KZ, 02UM37Z, 02UM3KZ, 02UM47Z, 02UM4KZ, 024G072, 024G082, 024G0K2, 024J072, 024J082, 024J0K2, 02RK07Z, 02RK0KZ, 02RK47Z, 02RK4KZ, 02RL07Z, 02RL0KZ, 02RL47Z, 02RL4KZ, 02U607Z, 02U608Z, 02U707Z, 02U708Z, 02U70KZ, 02U737Z, 02U738Z, 02U73KZ, 02U747Z, 02U748Z, 02U74KZ, 02UK0KZ, 02UK3KZ, 02UK4KZ, 02UL0KZ, 02UL3KZ, 02UL4KZ, 02Q50ZZ, 02Q53ZZ, 02Q54ZZ, 02QM0ZZ, 02QM3ZZ, 02QM4ZZ, 02QB0ZZ, 02QB3ZZ, 02QB4ZZ, 02QC0ZZ, 02QC3ZZ, 02QC4ZZ, 02BK0ZZ, 02NH0ZZ, 02RM0JZ, 02RP0JZ, 02RQ0JZ, 02RR0JZ, 02170ZP, 02170ZQ, 02170ZR, 02LS0ZZ, 02LT0ZZ, 02RM0JZ, 02U70JZ, 024F07J, 024F08J, 024F0JJ, 024F0KJ, 02S00ZZ, 02S10ZZ, 021L0ZW, 021L4ZW  **CPT:** 33120, 33130, 33300, 33305, 33310, 33315 | Within 30 days before index AF diagnosis |
| **Cardiac surgery:** valve repair or replacement surgery | **ICD-10-PCS:** 02RF07Z, 02RF08Z, 02RF0JZ, 02RF0KZ, 02RF37H, 02RF37Z, 02RF38H, 02RF38Z, 02RF3JH, 02RF3JZ, 02RF3KH, 02RF3KZ, 02RF47Z, 02RF48Z, 02RF4JZ, 02RF4KZ, 02RG07Z, 02RG08Z, 02RG0JZ, 02RG0KZ, 02RG37H, 02RG37Z, 02RG38H, 02RG38Z, 02RG3JH, 02RG3JZ, 02RG3KH, 02RG3KZ, 02RG47Z, 02RG48Z, 02RG4JZ, 02RG4KZ, 02RH07Z, 02RH08Z, 02RH0JZ, 02RH0KZ, 02RH37H, 02RH37Z, 02RH38H, 02RH38Z, 02RH3JH, 02RH3JZ, 02RH3KH, 02RH3KZ, 02RH47Z, 02RH48Z, 02RH4JZ, 02RH4KZ, 02RJ07Z, 02RJ08Z, 02RJ0JZ, 02RJ0KZ, 02RJ47Z, 02RJ48Z, 02RJ4JZ, 02RJ4KZ, 02UG3JZ, X2RF032, X2RF332, X2RF432  **CPT:** 33365, 33366, 33390, 33391, 33404-33406, 33410-33417, 33420, 33422, 33425-33427, 33430, 33440, 33463-33465, 33468, 33471, 33474, 33475 | Within 30 days before index AF diagnosis |

**Supplemental Table 3:** Medical claims codes used to define comorbid conditions

| **Condition** | **ICD-10-CM Codes** |
| --- | --- |
| Hypertension | I10: Essential (primary) hypertension I11: Hypertensive heart disease I12: Hypertensive chronic kidney disease I13: Hypertensive heart and chronic kidney disease I15: Secondary hypertension |
| Diabetes | E10: Type 1 diabetes mellitus  E11: Type 2 diabetes mellitus  E13: Other specified diabetes mellitus |
| Dyslipidemia | E78: Disorders of lipoprotein metabolism and other lipidaemias |
| Chronic Kidney Disease | N18: Chronic kidney disease |
| Chronic Obstructive Lung Disease | J42: Unspecified chronic bronchitis J43: Emphysema J44: Other chronic obstructive pulmonary disease |
| Peripherial Vascular Disease | I70.2: Atherosclerosis of arteries of extremities  I73: Other peripheral vascular diseases |
| Coronary Artery Disease | I20: Angina pectoris  I25: Chronic ischemic heart disease |
| Heart Failure | I50: Heart failure |
| Obstructive Sleep Apnea | G47.33: Obstructive sleep apnea |
| Ischemic Stroke | I63: Cerebral infarction |
| Myocardial Infarction | I21: Acute myocardial infarction  I22: Subsequent myocardial infarction |
| Aortic Stenosis | I35.0: Nonrheumatic aortic (valve) stenosis |
| Aortic Regurgitation | I35.1: Nonrheumatic aortic (valve) insufficiency |
| Mitral Regurgitation | I34.0: Nonrheumatic mitral (valve) insufficiency |
| Tricuspid Regurgitation | I36.1: Nonrheumatic tricuspid (valve) insufficiency |
| Gastrointestinal Hemorrhage | K25.0, K25.2, K25.4, K25.6: Gastric ulcer with bleeding  K26.0, K26.2, K26.4, K26.6: Duodenal ulcer with bleeding  K27.0, K27.2, K27.4, K27.6: Peptic ulcer with bleeding  K28.0, K28.2, K28.4, K28.6: Gastrojejunal ulcer with bleeding  K29.01: Acute gastritis with bleeding  I85.01: Esophageal varices with bleeding  K22.11: Ulcer of esophagus with bleeding  K92.0: Hematemesis  K92.1: Melena  K55.21: Angiodysplasia of colon with hemorrhage  K62.5: Hemorrhage of anus and rectum  K92.2: Gastrointestinal hemorrhage, unspecified |
| Intracranial Hemorrhage | I60: Nontraumatic subarachnoid hemorrhage  I61: Nontraumatic intracerebral hemorrhage  I62: Other and unspecified nontraumatic intracranial hemorrhage |

**Supplemental Table 4:** Medical claims codes used to define the procedures used in the AF patient cohort

| **Procedure** | **Codes** |
| --- | --- |
| Electrical Cardioversion | **CPT:** 92960 |
| Pulmonary Vein Ablation | **CPT:** 93656 **ICD-10-PCS:** 025S0ZZ, 025S3ZZ, 025S4ZZ, 025T0ZZ, 025T3ZZ, 025T4ZZ |
| Atrioventricular Node Ablation | **CPT:** 93650 **ICD-10-PCS:** 02580ZZ, 02583ZZ, 02584ZZ |
| Left Atrial Appendage Occlusion | **CPT:** 33340, 0281T |
